# Effects of Contact‐Heat Stimulation Intensity on the Reliability of Subjective Pain Rating of Individuals With Different Heat Tolerance

**DOI:** 10.1155/prm/3402006

**Published:** 2026-02-25

**Authors:** Sam C. C. Chan, Tom C. W. Tsoi

**Affiliations:** ^1^ Applied Cognitive Neuroscience Laboratory, Department of Rehabilitation Sciences, The Hong Kong Polytechnic University, Hong Kong, China, polyu.edu.hk

## Abstract

**Background:**

Studies revealed that contact‐heat stimulations mediate pain perception due to temporal summation of second pain (TSSP). How heat intensity affects the reliability of the pain rating of individuals with different heat tolerance is not well examined. This study investigated (1) the influence of the preceding contact‐heat stimuli with different levels of intensity on the reliability of subjective pain rating and (2) the differences in reliability of subjective pain rating of participants with high and low sensory sensitivity or heat tolerance.

**Method:**

Participants with intact sensory function were divided into (1) high (*n* = 17) and low (*n* = 13) sensitivity groups based on the cutoff temperature of 42°C or (2) high (*n* = 18) and low (*n* = 12) pain‐tolerance groups based on the cutoff temperature of 47°C equivalent to numerical rating scale (NRS) of 7. In each trial, participants were given a pair of 2‐s contact‐heat stimuli (an interstimulus interval of 2.5 s) at the left thenar eminence and were asked to report an NRS rating. Four blocks of intensity combinations were given: Low–Low, High–High, Low–High, and High–Low conditions, with 72 trials in each block.

**Results:**

Findings revealed that high heat‐tolerance group results had lower intraclass correlation coefficients (ICCs) when contact‐heat stimuli were preceded by another with higher intensity (ICC = 0.551–0.747) compared to those preceded by lower intensity (ICC = 0.724–0.818). In contrast, the ICCs of the low heat‐tolerance group were found to be relatively higher regardless of heat intensity (ICC = 0.595–0.806).

**Conclusions:**

The TSSP effect reflected by lower pain rating reliability appears to be induced in the high heat‐tolerance group when a contact‐heat stimulation is preceded by another stimulation with higher intensity but with the same duration. This is possibly due to the longer offset time of contact‐heat stimulations with higher intensity, and also the top‐down modulatory effects in this high heat‐tolerance group. Further electrophysiological studies would be needed to investigate the underlying neural processes of TSSP in individuals with different heat tolerance.

## 1. Introduction

### 1.1. Introduction of TSSP and Mechanisms

Noxious contact heat is regarded as a pertinent type of stimulation to study nociceptive pain [1, 2]. Thus, it is important to understand the physiological and cognitive characteristics of the stimulus. A number of studies have examined the phenomenon of the temporal summation of second pain (TSSP) induced by repeated noxious contact‐heat stimuli to C‐fibre nociceptors in a repetitive and phasic mode [3–5]. This, in turn, influences pain perception across time. C‐fibres convey noxious heat signals from the periphery to the cortex with a slower speed of 0.25–0.15 ms^−1^ compared to the faster speed of 2–30 ms^−1^ of Aδ fibres, which contribute to the affective component of pain perception [6, 7]. When noxious stimuli are repetitively applied, the fibres in the first‐order neuron deliver nociceptive stimuli to the cell bodies inside the dorsal root ganglion. Due to the slow onset and offset of C‐fibres, the nociceptive signals would be intensified with consecutive signals, leading to central sensitisation in dorsal horn neurons. Previously, studies examined the optimal interstimulus intervals (ISIs) that would evoke the effect of TSSP [3, 8, 9]. Vierck et al. [9] manipulated ISIs to control the activation of nociceptors that may cause temporal summation. Five different thermode temperatures with 4 different ISIs (3s, 4s, 5s and 6s) were repetitively applied on the participant’s thenar. A moderate degree of temporal summation was found at an ISI of 3 s, and the least temporal summation was obtained at an ISI of 6 s. How different levels of contact heat would affect the stability of individuals with different sensory thresholds or heat tolerance was not readily addressed. This study aimed to examine the phenomenon of temporal summation under a relatively short stimulus duration and an ISI of 2 s of contact heat with lower and higher temperatures.

Recent studies have revealed that a higher intensity of contact heat with the same ISI may induce a stronger tendency of TSSP [9, 10]. Previous studies have applied a range of relatively high temperatures of contact heat stimulus that may induce TSSP, including 45°C–53°C [9], 47°C–51°C and 44°C–48°C [1, 11]. Few attempts, however, have been made to examine how contact heat with different levels of intensity would affect the reliability of subjective pain rating under the influence of TSSP. According to Vierck et al. [9], the effect of TSSP was not conspicuous in healthy participants with lower heat tolerance. In contrast, the phenomenon appeared to be elicited in those who demonstrated higher pain tolerance. This suggests that individual heat tolerance may have a longer offset time and thus accumulate more strongly across consecutive stimuli, leading to a more pronounced TSSP effect. When the perceptual signal is rapidly escalating due to TSSP, participants may need to continually recalibrate their internal reference for pain intensity, which can reduce the stability of their subjective ratings. In this context, the reliability of pain ratings—largely governed by top‐down cognitive processes such as attention, appraisal and perceptual anchoring—may be compromised when TSSP is strong under relatively high contact‐heat stimulation. Furthermore, it is speculated that individuals with higher levels of heat tolerance may require higher demand in top‐down cognitive control over the incoming contact‐heat stimuli, influencing the stability of their ratings. The phenomenon has not been well addressed. Little is known about the reliability of pain perception under contact‐heat stimulations and how individual differences in heat tolerance associate with TSSP to affect the consistency of subject pain rating.

The aims of the study were twofold: (1) to investigate the influence of the preceding contact‐heat stimulus with fixed ISI of 2 s and different intensities on the reliability of pain perception as reflected by subjective pain rating and (2) to investigate the differences in reliability of pain perception on thermal sensation with different intensity of healthy participants with higher and lower sensory sensitivity and with high and low heat tolerance. The findings could provide insight into a suitable strategy and procedure to apply heat stimulations in clinical or experimental contexts.

## 2. Materials and Methods

### 2.1. Study Design

The participants who fulfilled the inclusion criteria were first asked to undergo a sensory determination procedure to determine contact‐heat temperature related to nociceptive threshold and moderate painful perception. Following this, they were given a series of contact‐heat stimuli with either higher or lower intensity. Each stimulus was preceded by higher and lower heat intensity with a fixed ISI of 2 s to simulate the TSSP effect, as this heat stimulus with an ISI of 3 s or less was shown to give the optimal TSSP [3, 8, 9]. At the end of each trial, the participant was required to give a subjective pain rating based on the numerical rating scale (NRS). The participants were divided into low and high heat‐tolerance groups based on the temperature of contact‐heat stimuli at < 47°C or ≥ 47°C, respectively, which were rated as the highest tolerable pain, equivalent to an NRS of 7.

### 2.2. Design and Participants

Thirty volunteering pain‐free participants were recruited through convenience sampling. The inclusion criteria were as follows: (1) age of 18 or above, (2) ability to communicate in Cantonese, (3) with a tertiary education or above and (4) having intact tactile and thermal functions. Those who had any skin lesions during the date of the experiment, a history of any diseases leading to chronic pain and nerve impairment of the upper limbs, cognitive deficits and language problems were excluded from the study. Among those joining the study, 18 participants were male and 12 were female, with a mean age of 21.4 (SD = 2.1 years). Each participant had a tertiary education or above and was Cantonese‐speaking. The joining participants were formed into two groups based on their sensory sensitivity and heat tolerance to examine whether these are associated with pain rating reliability. Participants who reported a contact‐heat stimulus with a temperature of < 42°C and ≥ 42°C as pain threshold were grouped as high‐sensitivity and low‐sensitivity groups, respectively [7]. On the other hand, participants who rated a contact‐heat stimulus with a temperature of < 47°C and ≥ 47°C as the highest tolerable pain equivalent to NRS of 7 after the procedure of sensory threshold determination were grouped into low and high heat‐tolerance groups, respectively [1, 5, 11]. The experimental protocols were approved by the Departmental Research Committee.

### 2.3. Experimental Procedure

The experimental procedure consisted of three parts. First, each participant was given a written informed consent after the study and its aims were explained. The participants needed to complete a number of screening tests, including a static two‐point discrimination (2PD) test, State–Trait Anxiety Inventory (STAI), Pain Catastrophising Scale (PCS) and a Chinese version of the Stroop test. Under the guidance, the participant’s sensory thresholds were determined. After training on NRS rating and pre‐experimental trials, the participants were engaged in an experiment concerned with pain rating reliability.

### 2.4. Apparatus for Contact‐Heat Stimulation

Contact‐heat stimuli in phase mode were given to participants via the PATHWAY Pain & Sensory Evaluation System (Medoc Ltd.). The device has a round thermode of 27 mm diameter contacting a cutaneous area. The thermode consists of a heating thermofoil (Minco Products, Inc., Minneapolis, MN) covered with a 25‐μm layer of thermoconductive plastic (Kapton, thermal conductivity of 0.1–0.35 W/mK at 23°C). The PATHWAY system has a heating‐up rate of 70°C per second and a cooling‐down rate of 40°C per second, allowing a rapid and repetitive onset and offset delivery of phasic stimuli with different durations and interstimulus intervals [12].

### 2.5. Sensory Threshold Determination

Prior to the pain rating reliability experiment, each participant was asked to undergo a standardised procedure to determine sensory thresholds. The procedure was conducted in a distraction‐free room with a consistent ambient temperature of around 22°C [13]. Each participant was seated comfortably on an armchair with a back supported. The thermode was applied to the thenar of the left hand (dermatome C6) [14]. Each participant was instructed to use his or her right hand to press a mouse button on the table, while forearms rested on the table.

A sensory threshold for each participant was determined by the limits module of the CHEPS stimulus presentation module [15]. Two sensory thresholds were determined using a standardised procedure: (1) minimal painful threshold, i.e., the lowest temperature that was perceived as painful and (2) tolerable painful threshold, i.e., the temperature that was perceived as tolerable (equivalent to NRS 7). To determine the minimal painful threshold, the temperature at the thermode started at a baseline temperature of 32°C, and the temperature was ramped at a rate of 70°C/s. The participant was required to stop the rise in temperature by clicking the mouse button when he or she started to perceive the contact heat stimulus as painful. The minimal painful threshold was computed by averaging the three temperatures obtained. To determine the tolerable painful threshold, the thermode setup and temperature characteristics were the same except that the participant was instructed to stop the rise in temperature when the stimulus started to be perceived as intolerable. For safety reasons, the CHEP programme would automatically stop temperature rise if the participant did not click the mouse at 51°C. There were five trials of each type of threshold determination with a one‐minute intermission in between to minimise the temporal summation effect [4]. The temperature of each type of threshold is obtained by averaging the 5 temperatures obtained.

Seven levels of temperature equivalent to NRS 1 to 7 were obtained. The temperature related to NRS 8 was excluded to avoid overstimulating the same cutaneous area with relatively high temperature. Six levels of temperature equivalent to NRS 1 to 3 and NRS 5 to 7 were selected to represent ‘low intensity’ and ‘high intensity’, respectively. The temperature of NRS 4 was excluded for clear demarcation between the temperature of the low and high intensity of contact‐heat stimuli.

### 2.6. Experimental Paradigm for Pain Rating Reliability

Before the experiment for pain rating reliability, a pre‐experiment training session was provided to each participant. This enabled participants to become familiar with giving subjective pain ratings using an 11‐point NRS for pain perception on contact‐heat stimuli with the temperature equivalent to NRS 1 to 7 determined during the procedure of sensory threshold determination.

**FIGURE 1:**
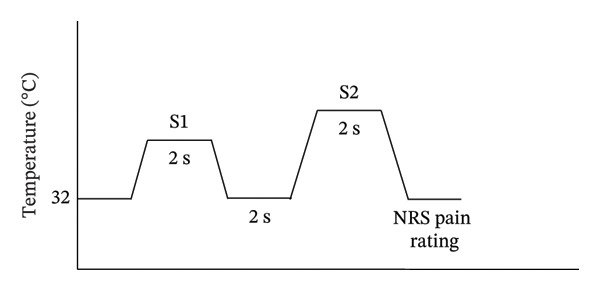
Experimental paradigm for pain rating reliability. In each trial, each participant was given a 2‐s thermal stimulus (S1) emitted from the CHEPS thermode (starting from a baseline temperature of 32°C and a ramp‐up rate of 40°C/s) with either low or high intensity, depending on the experimental condition. Another 2‐s thermal stimulus (S2) with either low or high intensity was then given to the participant 2 s after.

Each participant then participated in the pain rating reliability experiment with the four combinations of temperature intensity conditions: (1) Low–Low, (2) High–High, (3) Low–High, and (4) High–Low. In each trial, each participant was given a first 2‐s thermal stimulus emitted from the CHEPS thermode with a baseline temperature of 32°C and a ramp‐up rate of 40°C/s. The top temperature of the thermal stimulus was pseudorandomly selected from one of the lower temperature equivalent to individual pain NRS 1 to 3 (‘Low’ condition) or from one of the higher temperature equivalent to individual pain NRS 5 to 7 (‘High’ condition), depending on one of the four experimental conditions. After the first thermal stimulus had ramped down to the baseline temperature of 32°C, the second 2‐s thermal stimulus (a ramp‐up rate of 40°C/s) selected from one of the lower temperature equivalent to either NRS 1 to 3 or one of the higher temperature equivalent to either NRS 5 to 7 was emitted from the thermode to the participant’s left thenar area. When the temperature of the thermal stimulus had returned to the baseline temperature of 32°C, the participant was required to report verbally the pain NRS rating of the second thermal stimulus, which was recorded by the experimenter. The intertrial interval was 1 s. There were 9 trials in one block. Four blocks for each temperature combination condition occurred in a pseudorandomised manner. A total of 144 NRS ratings were given by each participant (Figure 1). All the blocks were randomised. Participants were given a 5‐min break after a block of 9 trials had been completed to avoid cognitive fatigue, or they could take breaks whenever they needed.

### 2.7. Pain Rating and Neuropsychological Tests

#### 2.7.1. NRS for Pain Perception

The NRS is used to measure the subjective rating of pain perception. It has an 11‐point scale with ‘0’ indicating ‘no pain’ and ‘10’ indicating ‘the worst imaginable pain’ [16]. The NRS was used in many studies to assess perception induced by obnoxious stimuli [17, 18]. A number of pain scales, such as the visual analogue scale (VAS) and NRS, have been established for pain perception for clinical or research use [19]. Gallasch and Alexandre [20] suggested that the NRS scale may be advantageous over VAS as it may not require as much cognitive demand. This would be particularly beneficial when applied to people who are less educated or the elderly. In addition, the reproducibility of NRS was found to be good to excellent with an ICC of 0.88 (95% CI = 0.81–0.92) [20].

#### 2.7.2. Static 2PD Test

The static 2PD function of hands was assessed by a two‐point discriminator [21]. A pair of two distance‐adjustable needles was placed on a certain cutaneous area supplied by one peripheral nerve. The procedure aims to measure the threshold of the smallest distance of separation of two needle points a respondent can detect [22]. In this study, static 2PD function was applied on the thenar eminence and thumb finger pulp of the left hand, covering the cutaneous distribution at C6 dermatome, as the thermode was placed in the area. In terms of psychometric properties, the test–retest reliability of static 2PD was shown to be satisfactory with an intraclass correlation coefficient (ICC) of 0.99 [23]. For the concurrent validity, its correlation was the function of object recognition and was also found to be good, with a correlation coefficient of 0.79 [15, 24].

#### 2.7.3. STAI

The STAI is used to measure trait and state anxiety [25]. It has 20 items to assess state (e.g., ‘I am tense; I am worried’) and trait aspects (e.g., ‘I am content; I am a steady person’) of anxiety separately. The respondent needs to rate each item based on a 4‐point scale in which a higher score represents greater anxiety. The trait version was shown to have excellent internal consistency (Cronbach’s alpha > 0.89) and excellent test–retest reliability (average *r* = 0.88) at multiple time intervals [26]. The Chinese version of STAI [27], used in this study, has been validated with satisfactory psychometric properties.

#### 2.7.4. PCS

The psychological aspect of one’s pain experience was measured by the PCS questionnaire [28, 29]. The instrument consisted of 13 items, and the respondent was required to rate based on a 5‐point self‐rating scale (‘0—not at all’ to ‘5—all the time’). Apart from the total scores, test items belong to three subscales: rumination, magnification and helplessness. The higher scores indicated a higher tendency of pain catastrophising. Good concurrent validity was supported by a moderate correlation of 0.59 between total PCS and the total inventory of negative thoughts in response to pain [30]. The reliability of the full version of PCS demonstrated a satisfactory internal consistency coefficient of 0.86 [31]. The Chinese version of PCS has been validated for Hong Kong clinical settings [32]. It was shown that the Chinese version of the instrument also had a satisfactory pain catastrophising level.

#### 2.7.5. Chinese Version of Stroop Test

The Chinese version of the Stroop test is associated with executive function for monitoring and solving conflict [33, 34]. The Chinese version was used in this study [35, 36]. Three parts are included: word reading (WR), colour naming (CN) and incongruent colour naming (INC). Each part consists of 100 stimuli on a 10 × 10 array. In the WR block, each respondent was shown with Chinese colour words printed in black (e.g., ‘紅’ [red], ‘藍’ [blue], ‘綠’ [green], ‘黃’ [yellow]). The respondent is required to read each word out quickly and accurately. In the CN block, each participant was presented with colour blocks printed on a 10 × 10 array on a white background and was required to name the colour blocks as fast and accurately as possible. In the INC block, the colour words were printed with incongruent ink colours (e.g., ‘blue’ printed in yellow, green or red). The respondent was required to name the ink colour of each word rather than the pronunciation of the word, as fast and accurately as possible. The number of errors, self‐corrected errors and time taken in each section were recorded by the test administrator. The overall test–retest reliability of the Stroop test was demonstrated to be good, with the test–retest reliability coefficient of 0.83 for the WR score, 0.74 for the colour reading score and 0.67 for the ICN score [37].

### 2.8. Statistical Analysis

Descriptive statistics (means and standard deviations) were produced for the cognitive and psychological measures, including STAI‐trait and state, PCS and Stroop test. Independent *t*‐tests were used to test the differences in the high‐ and low‐tolerance groups’ performances. The level of statistical significance was set at *p* < 0.05. Means and standard deviations of the temperature of critical thresholds, i.e., minimal painful and tolerable painful thresholds, of the two tolerance groups were also obtained. For achieving the study objectives, a measure of test–retest reliability of the NRS rating on pain perception was taken with a second stimulus computed for the high and low heat‐tolerance groups by using ICC (2,1) (i.e., two‐way random, single measure with 95% confidence intervals). According to Cicchetti [38], ICC values from 0.00 to 0.40, 0.41 to 0.59, 0.60 to 0.74 and 0.75 to 1.00 will be categorised as ‘poor’, ‘fair’, ‘good’ and ‘excellent’, respectively. All statistical tests were computed by using the software SPSS 24 (SAS Institute, Cary, NC, USA).

## 3. Results and Discussion

### 3.1. Performances in Sensory and Neuropsychological Tests and Painful Threshold Determination

Based on the painful threshold at the temperature of < 42°C or ≥ 42°C, 17 and 13 participants were categorised into high sensory sensitivity and low‐sensitivity groups, respectively. Based on the critical temperature for pain tolerance suggested in previous studies [1, 39], 18 and 12 participants were categorised into high heat‐tolerance (NRS of 7 ≥ 47°C) and low heat‐tolerance (NRS of 7 < 47°C) groups, respectively. The scores in sensory and neuropsychological tests of the participants in the two sensory sensitivity groups and two tolerance groups are summarised in Table 1. Independent t‐tests showed no significant differences in these tests (*p* < 0.05). It was shown that the two groups of participants had intact 2PD functions. STAI‐Trait did not reveal depression trait, and PCS scores did not suggest a tendency towards pain catastrophising. The executive function for conflict resolution was found to be within a normal level as reflected by the Stroop test.

**TABLE 1:** Participants’ scores in sensory and neuropsychological tests.

Scale	HST	LST	LHT	HHT
	Mean (SD)	Mean (SD)	Mean (SD)	Mean (SD)
2PD test	6.8 (1.4)	7.2 (2.1)	6.7 (1.5)	7.4 (2.7)
STAI‐State	37.0 (5.2)	35.9 (7.3)	37.0 (7.6)	36.4 (7.8)
STAI‐Trait	45.1 (5.5)	43.9 (5.8)	44.6 (6.7)	42.6 (5.0)
PCS	17.8 (8.8)	19.1 (9.2)	18.0 (9.9)	18.9 (10.2)
Chinese Stroop test: time interference score	30.2 (10.4)	43.7 (6.7)	31.9 (12.4)	42.6 (5.0)

*Note:*​ LHT = low heat‐tolerance group; 2PD = static two‐point discrimination. Time interference score = (score of colour‐word test) − (score of colour test + score of word test)/2.

Abbreviations: HH Group, high heat‐tolerance group; HS Group, High‐sensitivity group; LS Group, low‐sensitivity group; PCS, Pain Catastrophising Scale; SD, standard deviation; STAI, State‐Trait Anxiety Inventory.

The mean temperature for the minimal painful threshold and the tolerable painful threshold of the participants in the high‐sensitivity group were found to be 40.8°C (SD = 3.8°C) and 46.9°C (4.5°C), respectively. On the other hand, the mean temperatures of the two sensory thresholds of the participants in the low‐sensitivity group were found to be 43.4°C (SD = 4.0°C) and 47.9°C (4.1°C). The mean temperature for the minimal pain threshold of the participants in the high‐tolerance group was obtained to be 42.8°C (SD = 3.3°C), while the mean temperature for the tolerable painful threshold was 47.7°C (SD = 2.3°C). The mean temperature for the minimal painful threshold of the participants in the low heat‐tolerance group was found to be 40.0°C (SD = 2.7°C), while the mean temperature for the tolerable painful threshold was obtained to be 44.9°C (SD = 1.8°C).

### 3.2. Reliability of Subjective Pain Rating in the High‐Sensitivity Group

The ICC (2‐way random‐effects model and single measure) revealed the reliability of subjective pain rating using NRS in the high‐sensitivity group to range from (Table 2). The values of ICC of the NRS rating ranged from 0.702 (95% CI = 0.590–0.821) (NRS 1) to 0.787 (95% CI = 0.643–0.836) (NRS 2) when low heat stimulations were preceded by low heat stimulations, i.e., Low–Low condition. The values of the ICC of the NRS rating ranged from 0.765 (95% CI = 0.588–0.862) (NRS 5) to 0.774 (95% CI = 0.624–0.783) (NRS 6) under the Low–High condition. On the other hand, the ICC values of NRS rating for low heat stimulations preceded by high heat stimulations, i.e., High–Low condition, ranged from 0.695 (95% CI = 0.597–0.755) (NRS 1) to 0.757 (95% CI = 0.629–0.814) (NRS 3). The values of ICC were found to range from 0.699 (95% CI = 0.654–0.731) (NRS 5) to 0.792 (95% CI = 0.606–0.794) (NRS 7) under High–High condition. This suggested an overall good level of reliability (Table 2).

**TABLE 2:** Intraclass correlation coefficient (ICC) (95% confidence interval) of numerical rating scale in high‐sensitivity (minimal painful threshold < 42°C) (*n* = 17) and low‐sensitivity (minimal painful threshold ≥ 42°C) (*n* = 13) groups.

Condition	NRS	High‐sensitivity group	Low‐sensitivity group
		ICC (95% CI)	ICC (95% CI)
LL	1	0.702 (0.590–0.821)	0.744 (0.619–0.800)
	2	0.787 (0.643–0.836)	0.737 (0.655–0.796)
	3	0.768 (0.698–0.812)	0.783 (0.687–0.825)

LH	5	0.765 (0.588–0.862)	0.788 (0.663–0.801)
	6	0.774 (0.624–0.783)	0.782 (0.691–0.884)
	7	0.770 (0.693–0.880)	0.738 (0.695–0.780)

HL	1	0.695 (0.597–0.755)	0.695 (0.603–0.754)
	2	0.716 (0.658–0.769)	0.645 (0.599–0.558)
	3	0.757 (0.629–0.814)	0.685 (0.697–0.830)

HH	5	0.699 (0.654–0.731)	0.781 (0.670–0.854)
	6	0.702 (0.634–0.757)	0.724 (0.546–0.887)
	7	0.792 (0.606–0.794)	0.786 (0.650–0.876)

*Note:* LL = Low–Low condition; LH = Low–High condition; HL = High–Low condition; HH = High–High condition; NRS = Numerical Pain Rating Scale.

### 3.3. Reliability of Subjective Pain Rating in the Low‐Sensitivity Group

The ICC revealed the reliability of subjective pain rating using NRS in the low‐sensitivity group to range from (Table 3). The values of ICC of the NRS ranged from 0.744 (95% CI = 0.619–0.800) (NRS 1) to 0.783 (95% CI = 0.687–0.825) (NRS 3) when low heat stimulations were preceded by low heat stimulations, i.e., Low–Low condition. Furthermore, the values of the ICC of the NRS rating ranged from 0.738 (95% CI = 0.695–0.780) (NRS 7) to 0.788 (95% CI = 0.663–0.801) (NRS 5) under Low–High conditions. On the other hand, the ICC values of NRS rating for low heat stimulations preceded by high heat stimulations, i.e., High–Low condition, ranged from 0.645 (0.599–0.558) (95% CI = 0.472–0.804) (NRS 2) to 0.695 (95% CI = 0.603–0.754) (NRS 1). Furthermore, the values of ICC were found to range from 0.724 (95% CI = 0.546–0.887) (NRS 6) to 0.786 (95% CI = 0.650–0.876) (NRS 7) under High–High condition. This suggested that the reliability is generally satisfactory regardless of the levels of contact heat (Table 2).

**TABLE 3:** Intraclass correlation coefficient (ICC) (95% confidence interval) of numerical rating scale in high‐tolerance (NRS of 7 ≥ 47°C) (*n* = 18) and low‐tolerance (NRS of 7 < 47°C) (*n* = 12) groups.

Condition	NRS	High‐tolerance group	Low‐tolerance group
		ICC (95% CI)	ICC (95% CI)
LL	1	0.724 (0.580–0.860)	0.702 (0.519–0.877)
	2	0.757 (0.622–0.878)	0.705 (0.522–0.878)
	3	0.818 (0.705–0.912)	0.683 (0.496–0.867)

LH	5	0.725 (0.557–0.855)	0.767 (0.603–0.908)
	6	0.264 (0.134–0.481)	0.787 (0.631–0.917)
	7	0.801 (0.682–0.903)	0.738 (0.565–0.894)

HL	1	0.635 (0.472–0.804)	0.595 (0.393–0.819)
	2	0.706 (0.558–0.849)	0.245 (0.100–0.528)
	3	0.747 (0.609–0.873)	0.605 (0.407–0.824)

HH	5	0.551 (0.385–0.745)	0.781 (0.624–0.914)
	6	0.625 (0.463–0.797)	0.724 (0.546–0.887)
	7	0.662 (0.506–0.821)	0.806 (0.660–0.926)

*Note:* LL = Low–Low condition; LH = Low–High condition; HL = High–Low condition; HH = High–High condition; NRS = Numerical Pain Rating Scale.

### 3.4. Reliability of Subjective Pain Rating in the High‐Tolerance Group

**FIGURE 2:**
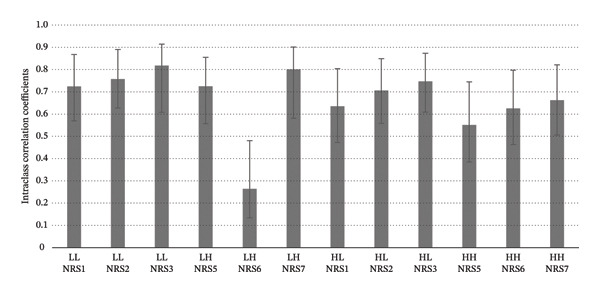
Intraclass correlation coefficients of subjective pain rating in the high heat‐tolerance group. Error bars denote 95% confidence interval.

The ICC revealed the reliability of the subjective pain rating using NRS in the high heat‐tolerance group to range from (Table 3). The values of ICC of the NRS rating ranged from 0.724 (95% CI = 0.580–0.860) (NRS 1) to 0.818 (95% CI = 0.705–0.912) (NRS 3) when low heat stimulations were preceded by low heat stimulations, i.e., Low–Low condition. The values of the ICC of the NRS rating ranged from 0.725 (95% CI = 0.557–0.855) (NRS 5) to 0.801 (95% CI = 0.68–0.903) (NRS 7) under Low–High conditions, suggesting good to excellent reliability regardless of the contact‐heat intensity (Cicchetti, 1994). There was an exception in that the ICC value was found to be fair for NRS 6 in Low–High condition, i.e., 0.264 (95% CI = 0.134–0.481). On the other hand, the ICC values of NRS for low heat stimulations preceded by high heat stimulations, i.e., High–Low condition, ranged from 0.635 (95% CI = 0.472–0.804) (NRS 1) to 0.747 (95% CI = 0.609–0.873) (NRS 3). The values of ICC were found to range from 0.551 (95% CI = 0.385–0.745) (NRS 5) to 0.662 (95% CI = 0.506–0.821) (NRS 7) under High–High condition. This suggested a fair to good level of reliability (Table 3 and Figure 2).

### 3.5. Reliability of Subjective Pain Rating in the Low‐Tolerance Group

**FIGURE 3:**
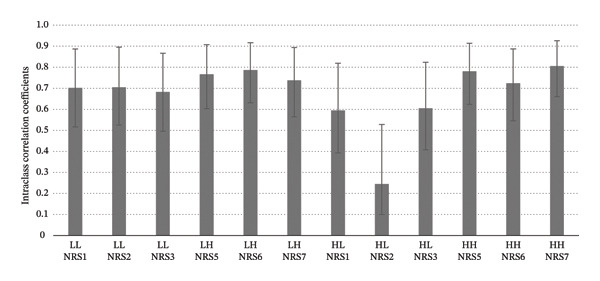
Intraclass correlation coefficients of subjective pain rating in the low heat‐tolerance group. Error bars denote 95% confidence interval.

The reliability pattern appeared to be different in the low heat‐tolerance group. The values of ICC were found to range from 0.683 (95% CI = 0.496–0.867) (NRS 3) to 0.702 (95% CI = 0.519–0.877) (NRS 1) when low heat stimulation preceded by low heat intensity, i.e., Low–Low conditions. Besides, the values of ICCs were found to range between 0.738 (0.565–0.894) (NRS 7) and 0.787 (95% CI = 0.631–0.917) (NRS 6) under Low–High conditions, suggesting a good reliability level. Besides, for low heat stimulations preceded by high heat intensity, i.e., High–Low condition, the ICC values obtained ranged from 0.595 (95% CI = 0.393–0.819) (NRS 1) to 0.605 (95% CI = 0.407–0.824) (NRS 3), suggesting fair reliability. The value of ICC of NRS 2 under the High–Low condition was found to be particularly low at 0.245 (95% CI = 0.100–0.528). Under High–High conditions, the values of ICC were revealed to range between 0.724 (0.546–0.887) (NRS 6) and 0.806 (95% CI = 0.660–0.926) (NRS 7). This suggested that the reliability appeared to be at a good to excellent level (Table 3 & Figure 3).

To sum up, the reliability of the NRS rating on high and low contact‐heat stimuli was generally found to be higher in the high heat‐tolerance group compared with the low‐tolerance group. Within the high‐tolerance group, the reliability of NRS rating appeared to be lowered when the heat stimuli were preceded by those with higher intensity compared with those with lower intensity. Such a trend was not as conspicuous in the low heat‐tolerance group.

## 4. Discussion

This study investigated the reliability of pain rating perception induced by different intensity levels of contact heat stimulation preceded by another stimulation with different intensity and examined how painful threshold or heat tolerance would affect the pain rating reliability. The results showed that the participants’ high and low heat tolerance would have a mediating effect on the reliability of their pain rating of different contact heat intensities. Such a mediating factor is not observed when grouping participants based on their sensitivity to the painful threshold. The main findings of this study were that the temporal summation effect was shown more conspicuously in the high‐tolerance group. The reliability of their pain perception in terms of ICC values on contact‐heat stimulations appeared to be lower when the stimulations were preceded by higher intensity (i.e., High–Low and High–High conditions) when compared with those with lower intensity (i.e., Low–Low and High–High conditions). Another observation was that the reliability of the NRS rating on contact heat stimuli tended to be lower within the low heat‐tolerance group, regardless of the heat intensity.

This study demonstrated that higher contact heat intensity with a fixed ISI of 2 s would induce a certain level of temporal summation as reflected by the lowered stability of subjective pain perception. The study conducted by Vierck et al. [9] suggested that temporal summation of phasic contact‐heat stimulus could be achieved by an ISI of 3 s or less and the onset latency could last for 2 s or more. According to these factors for temporal summation, it is speculated that the nociceptors under contact‐heat stimulation with higher intensity may have longer offset time to the resting state [4]. This might reduce the interstimulus interval with the subsequent stimulus, leading to an effect of temporal summation. The wind‐up effect from the primary nociceptor would lead to instability in pain perception as reflected by the NRS ratings on contact‐heat stimuli preceded by higher heat intensity (ICC = 0.551–0.747) when compared with those on lower heat intensity (ICC = 0.724–0.818) in the high heat‐tolerance group. As for the low heat‐tolerance group, such an instability of pain NRS rating for higher heat intensity was not as obvious. It could be possibly because the absolute temperature received by the low heat‐tolerance groups was not high relatively and the offset of the bottom‐up nociceptive signals was long enough to induce temporal summation.

The more obvious temporal summation found in the high heat‐tolerance group appears to be consistent with previous studies on the characteristics of temporal summation [4, 5, 9, 40]. It was found that temporal summation appeared to be more intensified when the temperature of the contact heat ranges between 45°C and 47°C. For example, in a recent study conducted by Eckert et al. [9], the temporal summation effect was found to be greater under intermittent contact stimulation when contact heat temperature of 45°C or above was applied either on hairy or glabrous skin. Another study conducted by Weissman‐Fogel and colleagues [5] revealed that the absolute temperature of contact‐heat stimulation, regardless of self‐perceived pain intensity, affected the magnitude of TSSP under a constant heat paradigm. Particularly among those with high heat tolerance, temperatures at or above 47°C would induce the intensification of the bottom‐up nociceptive signal to the higher cortical regions. Though further investigations are needed, it is speculated that top‐down modulation or adaptation of those with higher heat tolerance might be outweighed by bottom‐up, stimulus‐driven processes in the presence of high temperature or salient stimuli, leading to the instability of pain rating. Thus, it is reasoned that the participants in high heat‐tolerance group would give pain ratings with lower reliability when a higher contact‐heat stimulus was preceded by another higher one.

The distinctiveness of this study has been to examine the effect of intensity of the preceding contact‐heat stimuli on the perception of the subsequent one without altering the onset or offset latency or interstimulus interval. Other studies have investigated the temporal summation effect through manipulating the onset latency [8, 9] and offset latency [41]. Furthermore, a higher temporal summation effect would lead to instability of pain perception as indicated by less reliable pain rating, suggesting that the preceding contact heat with stronger intensity may have an effect to evoke temporal summation. The underlying mechanism of temporal summation affected by the preceding contact‐heat intensity could be explained by the change in the membrane potential of postsynaptic neurons [42, 43]. According to Mannion and Woolf [42], a higher postsynaptic potential was generated when more neurotransmitters bind with the receptors on the membrane of postsynaptic neurons of the nociceptors. Several studies have reported that action‐potential firing frequency at the postsynaptic terminal would increase inside the nociceptive neurons under higher contact‐heat intensity 45, 46]. Besides, Volgushev, Vidyasagar, Chistiakova and Eysel [44] also pointed out that there has been a positive correlation between excitatory postsynaptic potential amplitude and increasing temperature. Based on the evidence, the excitatory postsynaptic potential of preceding stimuli may stay longer in the postsynaptic neuron than the interstimulus interval when receiving the next subsequent stimuli, which would in turn prolong membrane excitation and intensify pain perception.

During the training prior to the experiment procedure, internal representations of different levels of contact heat intensity were supposed to be developed in the long‐term storage of the participant’s mind [45]. When an individual tries to rate a nociceptive stimulation after receiving it at the cortical level, he or she needs to disengage from the external nociceptive stimulus, shift attention to the internal representations retrieved from long‐term storage and re‐engage the external stimuli to discriminate the level of pain perception [45]. When an individual re‐engages the stimuli externally for comparison with the corresponding internal representation for rating the pain perception, mental effort would be required when comparing the internal representations of thermal stimulations with low salience [45]. This may lead to a less stable perception of the external stimuli which in turn affects the reliability of subjective pain rating. This phenomenon would be expected to be accentuated in the high heat‐tolerance group when the contact heat stimuli were preceded by the one with high intensity (i.e., High–Low and High–High conditions). Based on the premise that higher temperature will lead to higher bottom‐up temporal summation effect, the NRS of 7 pain perception of High–High condition should have the lowest ICC when compared to other NRS levels when preceded by a low heat intensity (Low–Low and Low–High) in the high heat‐tolerance group. However, the ICC of NRS of 7 pain perception was not the lowest. This suggests that the reliability of subjective pain rating was not merely affected by the bottom‐up temporal summation effect. A top‐down approach may also affect the reliability of subjective pain rating due to the salience of thermal stimulations. NRS 7 was the highest corresponding temperature received by the participants which created high salience of the thermal stimulation. Stimulations with higher salience may require fewer mental efforts when the participants matched the internal representations to the pain perception felt from external stimuli in the re‐engaging process, which may lead to a higher chance of correct perception. This may result in higher reliability of pain rating, thus higher reliability of NRS 7 in High–High condition.

In contrast to the high heat‐tolerance group, the reliability of subjective pain rating of the low heat‐tolerance group was found to be higher. This suggests that the reliability of subjective pain rating was less affected by the temporal summation effect. In the low heat‐tolerance group, the highest temperature of contact heat stimuli they received was below 47°C, whereas in the high heat‐tolerance group, the highest temperature of contact heat stimuli they received was above 47°C. This result is in line with the findings of Weissman‐Fogel, Drorand and Defrin [5] that 47°C is the temperature which initiates pain intensification. This suggests that contact heat stimulation at 47°C is the level of temperature that may evoke temporal summation in healthy participants. It is noteworthy that the ICCs of pain rating were relatively lower for the NRS rating of 2 in the High–Low condition of the low heat‐tolerance group and the NRS rating of 6 in Low–High condition of the high heat‐tolerance group. This suggested that the reliability of the NRS rating of a contact‐heat stimulus seemed to be undermined when it was preceded by another stimulus with different types of intensity, i.e., High–Low or Low–High and with a tolerance level different from stimulus. It is speculated that NRS of 2 and 6 may not have a clear mental anchor on the scale for perceptual rating, and the preceding contact‐heat stimuli, which are of different tolerance levels, may require the participants to put extra mental effort to perceive the intensity, leading to lower reliability of the subsequent stimulus. Further electrophysiological studies would be needed to allow a better understanding of the interference phenomenon.

The results of this study provide insights for applying contact‐heat stimulation during sensory testing, such as quantitative sensory tests [46]. Clinically, hot and cold discrimination kits are commonly used to assess the ability of neurological deficits in patients to discriminate temperature, which is an important factor for protective touch [47]. The administration of this assessment involves the application of contact heat stimuli to the evaluatee’s skin. The reliability of this thermal discrimination assessment may be influenced by the temporal summation effect. When thermal stimulus of the probe for hot temperature is applied to the evaluatee’s skin, temporal summation may be evoked if the duration of the probe applied is too long, the temperature is too high, or the interstimulus interval is too short. These factors will affect the reliability of the assessment results. This study provides insight for the evaluator to obtain more accurate thermal discrimination assessment results.

The possibility that our results were influenced by the top‐down processes is worth considering. The attention and rehearsal of internal representations of high‐ and low‐salience thermal sensations, which were associated with the mental conflicts in the re‐engaging process, may influence the reliability of subjective pain rating. This study, however, did not focus on these top‐down processes by monitoring the evoked potential in the neural pathways involved in these brain activities. As a result, some possible interactions between the bottom‐up temporal summation effect and top‐down attention processes were not captured in this study. Besides, though the pattern of reliability of pain rating of same groups of participants with different levels of sensitivity or tolerance appeared to be dependent on each other, future studies could be conducted to clarify whether the phenomenon of heat sensibility and tolerance are related to the neurological process using neurophysiological methods. The quantitative methods could confirm whether top‐down regulation is being overloaded by high‐intensity stimulation. As the study only requires participants to give subjective ratings on pain perception, it is worthwhile to consider the reliability of pain rating that would be different between men and women, considering the latter appears to have higher tolerance. Besides, this study only used an experimental paradigm with a fixed duration of contact‐heat stimuli with constant ISI. The different timing combinations of the stimuli and the ISI could be adopted in the paradigm in future studies. This would further enhance the applicability of the study findings. Another limitation of this study is the lack of comparison of the results with the patient group. As mentioned previously, the TSSP effect may influence the reliability of thermal discrimination assessment, which is generally used among patients with neurological deficits. The previous study by Potvin, Paul‐Savoie, Morin, Bourgault, and Marchand [43] pointed out that temporal summation effects are more prominent in fibromyalgia patients when compared to healthy participants. Since only healthy participants with a relatively small sample size (*n* = 30) were recruited in the present study, this limits the generalisability to other groups, such as individuals with chronic pain.

## 5. Conclusion

This study showed that preceding contact heat stimuli with higher contact heat intensity had an influence on the reliability of pain perception on thermal sensation as reflected by subjective pain rating in the high heat‐tolerance group. Moreover, the findings suggest that 47°C was the level of temperature that could evoke temporal summation in healthy participants. This study may be beneficial to the prescription of pain‐related assessments, especially in enhancing the accuracy of assessments or designing experimental protocols associated with temporal summation for those with neurological deficits. However, the present results only reflect the temporal summation effect in the bottom‐up pain pathway and in healthy participants. Further studies may consider incorporating an electroencephalogram assessment to monitor the potential variations among the participants in top‐down attention processes. Relevant follow‐up research may focus on exploring the difference in temporal summation among healthy participants and patients.

## Author Contributions

All listed authors should have contributed to the manuscript substantially. The first author has made a substantial contribution to the concept or design of the article, and all authors have contributed to the acquisition, analysis or interpretation of data for the article.

## Funding

No funding sources supported the research work.

## Disclosure

All authors have agreed to the final submitted version.

## Ethics Statement

The study was approved by the Departmental Research Committee (reference no.: HSEARS20170614001) on 03 July 2017.

## Consent

All participants gave consent to participate in the study.

## Conflicts of Interest

The authors declare no conflicts of interest.

## Data Availability

The data that support the findings of this study are available upon request from the corresponding author. The data are not publicly available due to privacy or ethical restrictions.

## References

[1] Tran T. D. , Wang H. , Tandon A. , Hernandez-Garcia L. , and Casey K. L. , Temporal Summation of Heat Pain in Humans: Evidence Supporting Thalamocortical Modulation, Pain. (2010) 150, no. 1, 93–102, 10.1016/j.pain.2010.04.001, 2-s2.0-77953019741. 20494516 PMC2916061

[2] Zhu Y. J. and Lu T. J. , A multi-scale View of Skin Thermal Pain: from Nociception to Pain Sensation, Philosophical Transactions Series A, Mathematical, Physical, and Engineering Sciences. (2010) 368, no. 1912, 521–559, 10.1098/rsta.2009.0234, 2-s2.0-76949102651. 20047938

[3] Eckert N. R. , Vierck C. J. , Simon C. B. et al., Methodological Considerations for the Temporal Summation of Second Pain, The Journal of Pain. (2017) 18, no. 12, 1488–1495, 10.1016/j.jpain.2017.07.009, 2-s2.0-85028770358. 28801070 PMC5682202

[4] Eckert N. R. , Vierck C. J. , Simon C. B. , Cruz-Almeida Y. , Fillingim R. B. , and Riley I. , Testing Assumptions in Human Pain Models: Psychological Differences Between First and Second Pain, The Journal of Pain. (2017) 18, no. 3, 266–273, 10.1016/j.jpain.2016.10.019, 2-s2.0-85009446651. 27888117 PMC5337172

[5] Weissman-Fogel I. , Dror A. , and Defrin R. , Temporal and Spatial Aspects of Experimental Tonic Pain: Understanding Pain Adaptation and Intensification, European Journal of Pain. (2015) 19, 408–418. 25045086 10.1002/ejp.562

[6] Laurie L. E. , Neuroscience: Fundamentals for Rehabilitation, 2007, 4th edition, WB Saunders Company, USA.

[7] Dubin A. E. and Patapoutian A. , Nociceptors: the Sensors of the Pain Pathway, Journal of Clinical Investigation. (2010) 120, no. 11, 3760–3772, 10.1172/jci42843, 2-s2.0-78049420335. 21041958 PMC2964977

[8] Koyama Y. , Koyama T. , Kroncke A. P. , and Coghill R. C. , Effects of Stimulus Duration on Heat Induced Pain: the Relationship Between real-time and Post-stimulus Pain Ratings, Pain. (2004) 107, no. 3, 256–266, 10.1016/j.pain.2003.11.007, 2-s2.0-0345829184. 14736588

[9] Vierck C. J.Jr, Cannon R. L. , Fry G. , Maixner W. , and Whitsel B. L. , Characteristics of Temporal Summation of Second Pain Sensations Elicited by Brief Contact of Glabrous Skin by a Preheated Thermode, Journal of Neurophysiology. (1997) 78, no. 2, 992–1002, 10.1152/jn.1997.78.2.992, 2-s2.0-1842336342. 9307129

[10] Gopalakrishnan R. , Machado A. G. , Burgess R. C. , and Mosher J. C. , The Use of Contact Heat Evoked Potential Stimulator (CHEPS) in Magnetoencephalography for Pain Research, Journal of Neuroscience Methods. (2013) 220, no. 1, 10–63, 10.1016/j.jneumeth.2013.08.015, 2-s2.0-84884555093. PMC3881311 23994044

[11] Staud R. , Weyl E. E. , Riley III J. L. , and Fillingim R. B. , Slow Temporal Summation of Pain for Assessment of Central Pain Sensitivity and Clinical Pain of Fibromyalgia Patients, PLoS One. (2014) 9, no. 2, 10.1371/journal.pone.0089086, 2-s2.0-84895860455. PMC3928405 24558475

[12] Granovsky Y. , Matre D. , Sokolik A. , Lorenz J. , and Casey K. , Thermoreceptive Innervation of Human Glabrous and Hairy Skin: a Contact Heat Evoked Potential Analysis, Pain. (2005) 115, no. 3, 238–247, 10.1016/j.pain.2005.02.017, 2-s2.0-19444385386. 15911150

[13] Wang L. , Gui P. , Li L. et al., Neural Correlates of heat-evoked Pain Memory in Humans, Journal of Neurophysiology. (2016) 115, no. 3, 1596–1604, 10.1152/jn.00126.2015, 2-s2.0-84984818534. 26740529 PMC4808118

[14] Paris T. , Misra G. , Archer D. , and Coombes S. , Effects of a Force Production Task and a Working Memory Task on Pain Perception, The Journal of Pain. (2013) 14, no. 11, 1492–1501, 10.1016/j.jpain.2013.07.012, 2-s2.0-84887028638. 24055565

[15] Riley J. L. , Cruz-Almeida Y. , Glover T. L. et al., Age and Race Effects on Pain Sensitivity and Modulation Among Middle-Aged and Older Adults, The Journal of Pain. (2014) 15, no. 3, 272–282, 10.1016/j.jpain.2013.10.015, 2-s2.0-84896841749. 24239561 PMC4005289

[16] Ferreira-Valente M. , Pais-Ribeiro J. , and Jensen M. , Validity of Four Pain Intensity Rating Scales, Pain. (2011) 152, no. 10, 2399–2404, 10.1016/j.pain.2011.07.005, 2-s2.0-80053187232. 21856077

[17] Fraenkel L. , Falzer P. , Fried T. et al., Measuring Pain Impact Versus Pain Severity Using a Numeric Rating Scale, Journal of General Internal Medicine. (2012) 27, no. 5, 555–560, 10.1007/s11606-011-1926-z, 2-s2.0-84862553987. 22081365 PMC3326111

[18] Jensen M. P. , Karoly P. , and Braver S. , The Measurement of Clinical Pain Intensity: a Comparison of Six Methods, Pain. (1986) 27, no. 1, 117–126, 10.1016/0304-3959(86)90228-9, 2-s2.0-0022467080. 3785962

[19] Alghadir A. , Anwer S. , Anwar D. , and Nezamuddin M. , The Development and Validation of Hundred Paisa Pain Scale for Measuring Musculoskeletal Pain: a Prospective Observational Study, Medicine. (2015) 94, no. 29, 10.1097/md.0000000000001162, 2-s2.0-84942428960. PMC4603018 26200616

[20] Gallasch C. H. and Alexandre N. M. , The Measurement of Musculoskeletal Pain Intensity: a Comparison of Four Methods, Review Enfermation. (2007) 28, no. 2, 260–265. 17907648

[21] Dellon A. , The Moving Two-point Discrimination Test: Clinical Evaluation of the Quickly Adapting fiber/receptor System, Journal of Hand Surgery American. (1978) 3, no. 5, 474–481, 10.1016/s0363-5023(78)80143-9, 2-s2.0-0018225658. 568154

[22] Jeroschherold C. , Assessment of Sensibility After Nerve Injury and Repair: a Systematic Review of Evidence for Validity, Reliability and Responsiveness of Tests, Journal Hand Surgery Brase. (2005) 30, no. 3, 252–264, 10.1016/j.jhsb.2004.12.006, 2-s2.0-18144368122. 15862365

[23] Novak C. , Mackinnon S. , and Kelly L. , Correlation of Two-point Discrimination and Hand Function Following Median Nerve Injury, Annals of Plastic Surgery. (1993) 31, no. 6, 495–498, 10.1097/00000637-199312000-00003, 2-s2.0-0027142890. 8297078

[24] Dellon A. and Kallman C. , Evaluation of Functional Sensation in the Hand, Journal of Hand Surgery. (1983) 8, no. 6, 865–870, 10.1016/s0363-5023(83)80083-5, 2-s2.0-0021018066. 6643961

[25] Spielberger C. D. , Gorsuch R. L. , Lushene R. , Vagg P. R. , and Jacobs G. A. , Standardized Assessment Instruments in Psychology, Psychological Assessment. (1983) 6, 284–290.

[26] Barnes L. L. B. , Harp D. , and Jung W. S. , Reliability Generalization of Scores on the Spielberger State–Trait Anxiety Inventory, Educational and Psychological Measurement. (2002) 62, no. 4, 603–618, 10.1177/0013164402062004005, 2-s2.0-0036352790.

[27] Shek D. T. , The Chinese Version of the State‐Trait Anxiety Inventory: Its Relationship to Different Measures of Psychological Well‐Being, Journal of Clinical Psychology. (1993) 49, no. 3, 349–358. 8315037 10.1002/1097-4679(199305)49:3<349::aid-jclp2270490308>3.0.co;2-j

[28] Chaves J. F. and Brown J. M. , Spontaneous Cognitive Strategies for the Control of Clinical Pain and Stress, Journal of Behavioral Medicine. (1987) 10, no. 3, 263–276, 10.1007/bf00846540, 2-s2.0-0023223235. 3612783

[29] Rosenstiel A. K. and Keefe F. J. , The Use of Coping Strategies in Chronic Low Back Pain Patients: Relationship to Patient Characteristics and Current Adjustment, Pain. (1983) 17, no. 1, 33–44, 10.1016/0304-3959(83)90125-2, 2-s2.0-0020575984. 6226916

[30] Osman A. , Barrios F. X. , Kopper B. A. , Hauptmann W. , Jones J. , and O’neill E. , Factor Structure, Reliability, and Validity of the Pain Catastrophizing Scale, Journal of Behavioral Medicine. (1997) 20, no. 6, 589–60, 10.1023/a:1025570508954, 2-s2.0-0031439683. 9429990

[31] Granot M. and Ferber S. , The Roles of Pain Catastrophizing and Anxiety in the Prediction of Postoperative Pain Intensity, The Clinical Journal of Pain. (2005) 21, no. 5, 439–445, 10.1097/01.ajp.0000135236.12705.2d, 2-s2.0-24144469771. 16093750

[32] Yap J. , Lau J. , Chen P. et al., Validation of the Chinese Pain Catastrophizing Scale (HK-PCS) in Patients with Chronic Pain, Pain Medicine. (2008) 9, no. 2, 186–195, 10.1111/j.1526-4637.2007.00307.x, 2-s2.0-39649096042. 18298701

[33] Stuss D. T. , Floden D. , Alexander M. P. , Levine B. , and Katz D. , Stroop Performance in Focal Lesion Patients: Dissociation of Processes and Frontal Lobe Lesion Location, Neuropsychologia. (2001) 39, no. 8, 771–786, 10.1016/s0028-3932(01)00013-6, 2-s2.0-0035016521. 11369401

[34] Wiech K. , Ploner M. , and Tracey I. , Neurocognitive Aspects of Pain Perception, Trends in Cognitive Sciences. (2008) 12, no. 8, 306–313, 10.1016/j.tics.2008.05.005, 2-s2.0-47249097422. 18606561

[35] Chan S. , Chan C. , Kwan A. , Ting K. , and Chui T. , Orienting Attention Modulates Pain Perception: an ERP Study, PLoS One. (2012) 7, no. 6, 10.1371/journal.pone.0040215, 2-s2.0-84863098537. PMC3387012 22768257

[36] Liu X. , Qi H. , Wang S. , and Wan M. , Wavelet-Based Estimation of EEG Coherence During Chinese Stroop Task, Computers in Biology and Medicine. (2006) 36, no. 12, 1303–1315, 10.1016/j.compbiomed.2005.08.002, 2-s2.0-33749119520. 16289018

[37] Franzen M. , Tishelman A. C. , Sharp B. H. , and Friedman A. G. , An Investigation of the test-retest Reliability of the Stroop Colorword Test Across Two Intervals, Archives of Clinical Neuropsychology. (1987) 2, no. 3, 265–272, 10.1093/arclin/2.3.265. 14589618

[38] Cicchetti D. V. , Guidelines, Criteria, and Rules of Thumb for Evaluating Normed and Cortex in Working Memory: Examining the Contents of Consciousness, Philosphy Transaction R Social Londen B B Biology Science. (1994) 353, 1819–1828.

[39] Kong J. T. , Johnson K. A. , Balise R. R. , and Mackey S. , Test-Retest Reliability of Thermal Temporal Summation Using an Individualized Protocol, The Journal of Pain. (2013) 14, no. 1, 79–88, 10.1016/j.jpain.2012.10.010, 2-s2.0-84871897654. 23273835 PMC3541942

[40] Hashmi J. A. and Davis K. D. , Effect of Static and Dynamic Heat Pain Stimulus Profiles on the Temporal Dynamics and Interdependence of Pain Qualities, Intensity, and Affect, Journal of Neurophysiology. (2008) 100, no. 4, 1706–1715, 10.1152/jn.90500.2008, 2-s2.0-57349146670. 18701756

[41] Lundy-Ekman L. , Neuroscience-E-Book: Fundamentals for Rehabilitation, 2013, Elsevier Health Sciences.

[42] Podivinský F. , Effect of Stimulus Intensity on the Rising Phase of the Nerve Action Potential in Healthy Subjects and in Patients with Peripheral Nerve Lesions, Journal of Neurology Neurosurgery and Psychiatry. (1967) 30. 10.1136/jnnp.30.3.227 PMC496173 4291840

[43] Janal M. N. , Raphael K. G. , Cook D. B. , Sirois D. A. , Nemelivsky L. , and Staud R. , Thermal Temporal Summation and Decay of after-sensations in Temporomandibular Myofascial Pain Patients with and Without Comorbid Fibromyalgia, Journal of Pain Research. (2016) 9, 641–652, 10.2147/jpr.s109038, 2-s2.0-84988918483. 27672341 PMC5026221

[44] Peng J. , Chan S. C. C. , Chau B. K. H. , Yu Q. , and Chan C. C. H. , Salience of Review Somatosensory Stimulus Modulating External-to-Internal Orienting Attention, Frontiers in Human Neuroscience. (2017) 11, 1–10. 28970787 10.3389/fnhum.2017.00428 PMC5609543

[45] Magerl W. , Krumova E. K. , Baron R. , Tölle T. , Treede R. D. , and Maier C. , Reference Data for Quantitative Sensory Testing (QST): Refined Stratification for Age and a Novel Method for Statistical Comparison of Group Data, Pain. (2010) 151, no. 3, 598–605, 10.1016/j.pain.2010.07.026, 2-s2.0-77957140470. 20965658

[46] Ceynowa M. , Tomasz M. , Pankowski R. , Rocławski M. , and Treder M. , The Thermal Sensitivity Test in Evaluating Outcome After Peripheral Nerve Injury, BioMed Research International. (2015) 2015, 1–9. 10.1155/2015/528356 PMC4493271 26199942

[47] Potvin S. , Paul-Savoie E. , Morin M. , Bourgault P. , and Marchand S. , Temporal Summation of Pain is Not Amplified in a Large Proportion of Fibromyalgia Patients, Pain Research Treatment. (2012) 2012, 938595–938596, 10.1155/2012/938595, 2-s2.0-84873869783. 22701791 PMC3372092

[48] Mannion R. J. and Woolf C. J. , Pain Mechanisms and Management: a Central Perspective, The Clinical Journal of Pain. (2000) 16, no. S1, S144–S156, 10.1097/00002508-200009001-00006. 11014459

[49] Belmonte C. and Cervero F. , Neurobiology of Nociceptors, 1996, Oxford University Press.

[50] Cesare P. , Moriondo A. , Vellani V. , and McNaughton P. A. , Ion Channels Gated by Heat, Processing Natural Academic Science USA. (1999) 96, no. 14, 7658–7663, 10.1073/pnas.96.14.7658, 2-s2.0-0033529214. PMC33597 10393876

[51] Volgushev M. , Vidyasagar T. R. , Chistiakova M. , Yousef T. , and Eysel U. T. , Membrane Properties and Spike Generation in Rat Visual Cortical Cells During Reversible Cooling, The Journal of Physiology. (2000) 522, no. 1, 59–76, 10.1111/j.1469-7793.2000.0059m.x, 2-s2.0-0033969327. 10618152 PMC2269736

